# Synthesis of TiC@C-anatase/rutile@polyvinyl alcohol/xylan: a powerful photocatalyst for degradation of organic pollutant under visible light

**DOI:** 10.1098/rsos.220080

**Published:** 2022-08-24

**Authors:** Yahya Absalan, Mostafa Gholizadeh, Mohammad Reza Razavi, Zeynab Dastani, Anh Thi Ngoc Vu, Olga Kovalchukova

**Affiliations:** ^1^ Department of Chemistry, Faculty of Science, Ferdowsi University of Mashhad, Mashhad, IR Iran; ^2^ Department of chemistry, Georgia University, Athens, GA 30602, USA; ^3^ Environmental Analysis Laboratory, Southern Branch of Vietnam-Russia Tropical Center, 3/2 Street District 10, Ho Chi Minh City, Vietnam; ^4^ Department of Inorganic and Analytical Chemistry, Kosygin Russian State University (Technology, Design, Art), 33 Sadovnicheskaya Street, Moscow 117997, Russia; ^5^ General Chemistry Department, RUDN University, 6 Miklukho-Maklaya Street, Moscow 117198, Russia

**Keywords:** titanium carbide, nanomaterial, polymer composite, photocatalyst, visible light

## Abstract

In this study, a composite bearing titanium carbide (TiC), titanium dioxide (TiO_2_), polyvinyl alcohol and xylan (TiC@C-anatase/rutile@polyvinyl alcohol/xylan) was synthesized and applied as a photocatalyst for the degradation of bromophenol blue (BPB) solution through several steps. Nanostructure of TiC and TiO_2_ in the anatase and rutile phases was obtained through heat treatment of TiC at different times and temperatures (TiC@AR) which led to a reduction in energy bandgap from UV to visible light, in addition to the enhancement of the surface activity. After TiC@AR polymerization by xylan and polyvinyl alcohol and obtaining TiC@AR/PX, the energy bandgap reduced to IR range (52% of the sunlight) while showing an enhancement in the surface activity. The photocatalytic activity of the compounds was tested by studying the decomposition of BPB solution under visible light. The result illustrated the ability of TiC and TiC@AR to decrease the concentration of BPB after 150 min by 35% and 37%, respectively, while this reduction was 72% for TiC@AR/PX. Considering the effective parameters, the energy bandgap and the surface layer played key roles in photocatalytic degradation.

## 1. Introduction

Organic contamination of water resources by industrial, hospital and laboratory wastes is one of the serious environmental problems. Various approaches have been developed to combat these health problems, among which coagulation [1] and biological membrane filtration (activated carbon absorption) [2,3] can be mentioned. Despite their advantages such as cost-effectiveness, these methods suffer from drawbacks like the long process and toxic by-products and sludge [4].

Recently, the advanced oxidation process has been introduced as an effective approach for the treatment of contaminated water [5]. In this method, produced radicals convert harmful organic pollutants to nontoxic materials [6] such as CO_2_ and H_2_O [5]. Semiconductor photocatalysts have several advantages, making them superior to other agents [7,8]. They are used in various applications, including photocatalytic water splitting (H_2_ evolution) [9], CO_2_ reduction [10], pollutant degradation [11] and bacterial disinfection [12].

TiO_2_ has found increasing popularity due to its efficiency, ease of synthesis, low cost and toxicity, and biocompatibility [13]. Its efficiency can be further improved by enhancing its surface activity through reducing its size to the nanometric range. One of the most effective parameters in estimating the ability of a photocatalyst is its energy bandgap. However, titanium dioxide (TiO_2_) cannot absorb visible light due to its large bandgap. This system is only activated by UV light, which has limited its application. This limitation can be resolved by modifying a TiO_2_ nano-photocatalyst for instance through doping [14,15], grafting [16], solid solution, semiconductor coupling, floatability amelioration, immobilization and magnetic separation [17–28].

For overcoming the weak absorption of visible light and rapid recombination rate of photogenerated electron–hole pairs, it is possible to modify the surface TiO_2_ with organic polymers as a coating structure to improve interfacial charge transfer [29], by decreasing the LUMO level through reducing the energy bandgap and increasing the photocatalytic efficiency under visible light [30]. Besides, these polymers have several functional groups, promoting the structure reactivity towards organic substances, leading to a crosslinked compound with high photocatalytic efficiency. Surface modification can also enhance the hydrophilic area for participation in organic reactions and improve the degradation and absorption of pollutants even under visible light [31,32].

In this study, a novel method was developed to prepare a structure for photocatalytic purposes. In this regard, first, the crystalline structure of TiO_2_ (anatase and rutile phases) was used alongside titanium carbide (TiC) through a specific heat treatment regime. Then, polyvinyl alcohol was coated as the crosslinker; while xylan served as a template for immobilization of crystalline TiO_2_, leading to a second layer through the refluxing method. Finally, the photocatalytic activity of the composite was evaluated in terms of bromophenol blue (BPB) dye removal under visible light. The results showed a substance with a large surface activity, which can be easily activated under visible light to efficiently remove organic harmful substances.

## 2. Material and methods

### 2.1. Material characterizations

XRD patterns were obtained with a GNR Explorer advance using 1.541 Å (Cu-Ka) radiation. Thermogravimetric analysis (TGA) and differential scanning calorimetry (DSC) were carried out with a Netzsch STA 449 F3 Jupiter installation in air. The surface activity of the obtained samples was determined by BET analysis at 77 K with a micrometric Belsorp-mini II. To estimate the mesoporosity and pore size distribution, the Barrett–Joyner–Halenda (BJH) method was used. A TESCAN BRNO-Mira3 LMU was used for taking nanoscale images for morphological study. TEM images were taken through a LEO 912AB using ethanol as disperser. An AVATAR 370 FT-IR spectrometer was used to obtain the FT-IR spectra of the samples at room temperature in the range of 4000–400 cm^−1^. Diffuse reflection/transmittance spectroscopy (DRS/DTS) was carried out by Avantes (Avaspec-2048-TEC). A tube furnace, EX-1700-3 L, was used for heat treatment. Ultrasonication (CLEAN1-L06) was done during the synthesis procedure. The photocatalytic application was carried out by a 350 W xenon lamp (UV–visible light). The system was cooled by water circulation and the temperature was maintained at approximately 20°C. To study the concentration of the light-exposed solution, UV–visible spectroscopy was carried out in the range of 200–800 nm using a Cary-50 scan spectrophotometer (Varian). Cyclic voltammetry was carried out with a potentiostat ACM model GillAC. The high-resolution TEM (HRTEM) images were taken with an FEI instrument, model TEC9G20 (200 kV)

### 2.2. Material and reagents

All the major precursors, including TiC, polyvinyl alcohol and xylan, were purchased from Merck and Sigma-Aldrich and used as received without further purification.

### 2.3. Synthesis of TiC@C-anatase/rutile@ polyvinyl alcohol/xylan (TiC@AR/PX)

TiC@AR/PX was synthesized in two steps, including the synthesis of TiC@anatase/rutile (TiC@AR) and its modification by xylan and polyvinyl alcohol. First, the best condition for oxidizing TiC to obtain TiC@AR was investigated. TiC was placed in a tube furnace operating under air atmosphere to react with the oxygen of the air at various temperatures and times. Three different temperatures of 350, 400 and 450°C were considered at different times. First, TiC was oxidized at 350°C for 30 (**TiC_a_**), 60 (**TiC_b_**), 90 (**TiC_c_**), 120 (**TiC_d_**), 180 (**TiC_e_**) and 240 (**TiC_f_**) min. Based on the XRD pattern, no signals related to anatase and rutile appeared. Two other thermal regimes were set which were 400°C for 30 (**TiC_g_**) and 60 (**TiC_h_**) min and 450°C for 30 (**TiC_i_**) and 60 (**TiC_j_**) min. Finally, the heat treatment at 450°C for 60 min was considered as the best condition, so the label of **TiC_j_** was converted to **TiC@AR**.

The second step involved modifying the surface of the TiC@AR with xylan/polyvinyl alcohol to obtain TiC@AR/PX. One gram of polyvinyl alcohol was dissolved in 25 ml water; after mixing at 70°C for 30 min, the solution was added to the mixture of xylan, which was obtained at the same condition followed by mixing for 30 min at 70°C to obtain a solution (solution A). Then, 0.05 g TiC@AR was added to the sample under ultrasonication at 70°C for 30 min.

Finally, the obtained solution was refluxed at 65–70°C for 3 h (solution B). Then, 25 ml of a mixed solvent (20% H_2_O and 80% ethanol) was added to solution B for precipitation; it was then kept at room temperature for a day to be prepared for decantation. After decanting, the precipitate was washed two times with the solution of 20% H_2_O : 80% ethanol, followed by drying at 105°C.

### 2.4. Cyclic voltammetry analysis

Cyclic voltammetry was carried out by the usual three electrode system, including working electrode (glassy carbon electrode (*d* = 2 mm)), reference electrode (Ag/AgCl) and counter electrode (platinum wire). The electrolyte was made of 0.1 M NaOH. N_2_ was used for saturating the experiment's environment and it was purged 15 min before the experiment. The potential window was set between −0.4 and 1 V with a scan rate of 50 mV s^−1^. The plot is obtained based on the IUPAC method, which means it starts from left to right.

### 2.5. Photocatalytic degradation of bromophenol blue as an organic harmful target

To test the photocatalytic degradation of TiC@AR/PX (photocatalyst), BPB was chosen as the organic harmful phenolic compound. First, 5 × 10^−3^ mol of catechol was prepared in water (500 cm^3^). Then, 5 × 10^−2^ mol of the photocatalyst was added to the solution under stirring. The first step of stirring was carried out in absolute darkness. After 15 min, 3 ml of the solution was withdrawn as the blank sample. Then, a 350 W Xe lamp (with a UV cut-off filter of greater than 400 nm) was applied as a visible light source. The solution was stirred under the light source, and 3 ml of the solution was withdrawn in 15-min intervals; the experiment was set for 150 min to obtain 10 samples. The concentration of the samples was measured by a UV–visible spectrometer. This experiment was repeated for TiC and TiC@AR. Throughout the experiment, the temperature was set at 25°C using water circulation and the required oxygen was supplied by an oxygen tank.

## 3. Results and discussion

**Figure 1.**
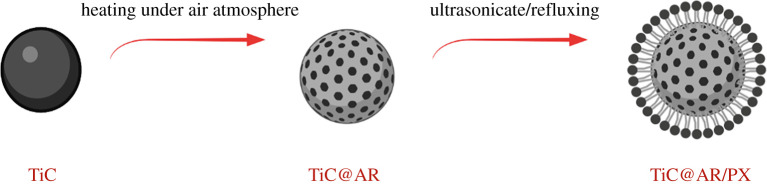
The formation process of TiC@AR/PX.

Figure 1 shows the procedure of the synthesis of TiC@AR/PX. By heat-treating TiC at different temperatures and times, it was converted to TiC@AR; then this product was converted to TiC@AR/PX through ultrasonication followed by refluxing.

**Figure 2.**
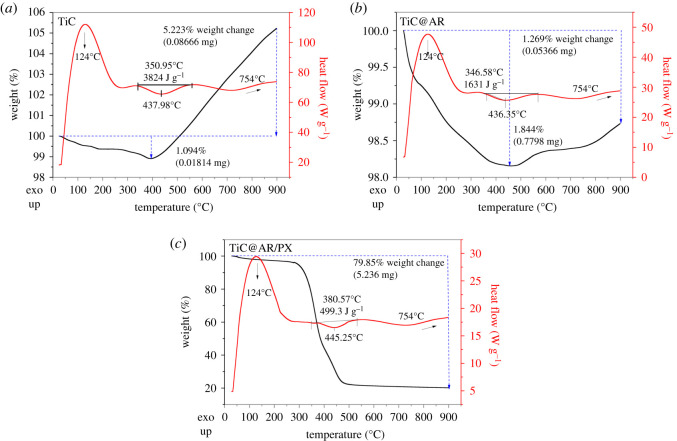
TGA patterns of the samples.

In the case of TiC, an initial weight loss occurred below 200°C due to evaporation of the interior water (figure 2). The reduction was continued at an almost constant rate at 200–400°C. This behaviour shows that the properties of TiC are constant at this temperature range. After that, a notable exothermic peak in the DSC curve (red line) indicated the weight gain due to the oxidation of TiC at a low exothermic rate leading to the formation of the second layer (TiO_2_) on the TiC surface. The slope of the TGA curve (black line) remained constant between 450°C and 900°C, suggesting that the overall composition of the substance is constant with no further changes. After 750°C, a slight increase can be detected in the DSC line due to CO_2_ release; however, the change is not significant and not very clear in the TGA curve [33].

The overall DSC is the same for TiC@AR and TiC@AR/PX since their organic compounds (in the case of TiC@AR/PX) have a lower melting point and they were decomposed at lower temperatures, leaving only TiC@AR. Indeed, in the case of TiC, it reacted with oxygen, giving rise to TiC@TiO_2_ (depending on the temperature, anatase, anatase/rutile and rutile phases). In the case of TiC@AR, oxygen is the only element reacting with TiC@AR; thus, the overall DSC line is again the same. TiC@AR/PX also has the same behaviour due to the mentioned explanation.

On the other hand, the TGA curve showed a better difference between the samples at different stages. The TGA line of TiC@AR shows the same behaviour as TiC up to 400°C with similar weight loss. A difference emerged between 400°C and 900°C. The weight gain after 400°C is due to the reaction with the oxygen leading to an exothermic peak. When the temperature reached 550°C, the slope of the TGA curve became milder as a result of phase transformation (probably to just rutile phase). The weight change was 1.84% which is negligible. In the case of TiC, however, the highest weight change was 5.22% (almost five times that of TiC@AR). It confirmed that TiC was converted to TiC@AR.

The most changes can be seen in the case of TiC@AR/PX. Similar to other samples, the behaviour of TiC@AR/PX was the same below 400°C (almost a 2% weight loss). However, after this temperature instead of an exothermic peak, an endothermic peak appeared, suggesting a decline in the content of the organic substance. The total weight change was about 80%, including 2% water (from 100 to 98%) and 78% of organic substance (polyvinyl alcohol and xylan). The residual weight remained constant between 500 and 900°C (i.e. TiC@AR).

**Figure 3.**
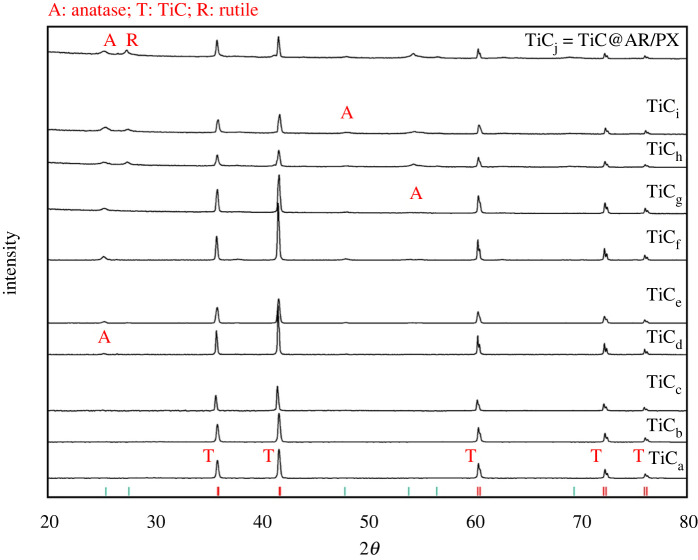
XRD patterns of different samples.

The structure of all samples was determined by XRD in the 2*θ* range of 20–80° (figure 3). The peaks corresponding to TiO_2_ appeared with enhancing temperature from 350°C to 450°C. TiC could not be properly oxidized at 350°C to reveal TiO_2_ peaks. JCPDS of TiC_a_ to TiC_e_ is the same (96-901-2565). According to the database, their crystalline structures are related to TiC (khamrabaevite). However, after 240 min of heating at 350°C (Ti_f_), a low-intensity peak corresponding to the anatase phase appeared (Ti_f_ = khamrabaevite: 96-901-2565, anatase: 96-900-9087).

With increasing temperature, the peaks related to both anatase and rutile phases appeared in TiC_g_, TiC_h_, TiC_i_ and TiC_j_ = TiC@AR (khamrabaevite: 96-901-2565, anatase: 96-900-9087, rutile: 96-900-9084). Based on the result, the best temperature and time for designing the phases of anatase/rutile on TiC was 450°C for 60 min (TiC@AR). The average size was calculated by the Scherrer equation $\left(D_{c}=k\lambda /\beta \cos\theta \right)$ (table 1).

**Table 1.** Chemical–physical features of the samples.

sample	2*θ*	crystallite size (nm)
TiC_a_	35.9, 41.68, 60.39, 72.28, 76.05	51.78
TiC_b_	35.9, 41.68, 60.39, 72.28, 76.05	79.31
TiC_c_	35.9, 41.68, 60.39, 72.28, 76.05	78.93
TiC_d_	35.9, 41.68, 60.39, 72.28, 76.05	54.28
TiC_e_	35.9, 41.68, 60.39, 72.28, 76.05	55.09
TiC_f_	25.18, 35.9, 41.68, 60.39, 72.28, 76.05	45.09
TiC_g_	25.18, 27.45, 35.91, 37.71, 41.27, 41.68, 47.94, 54.31, 54.89, 56.52, 60.39, 72.28, 76.05	39.70
TiC_h_	25.18, 27.45, 35.91, 37.71, 41.27, 41.68, 47.94, 54.31, 54.89, 56.52, 60.39, 72.28, 76.05	28.24
TiC_i_	25.18, 27.45, 35.91, 37.71, 41.27, 41.68, 47.94, 54.31, 54.89, 56.52, 60.39, 72.28, 76.05	36.01
TiC@AR	25.18, 27.45, 35.91, 37.71, 41.27, 41.68, 47.94, 54.31, 54.89, 56.52, 60.39, 72.28, 76.05	34.96
TiC@AR/PV	25.18, 27.45, 35.91, 37.71, 41.27, 41.68, 47.94, 54.31, 54.89, 56.52, 60.39, 72.28, 76.05	20.50

**Figure 4.**
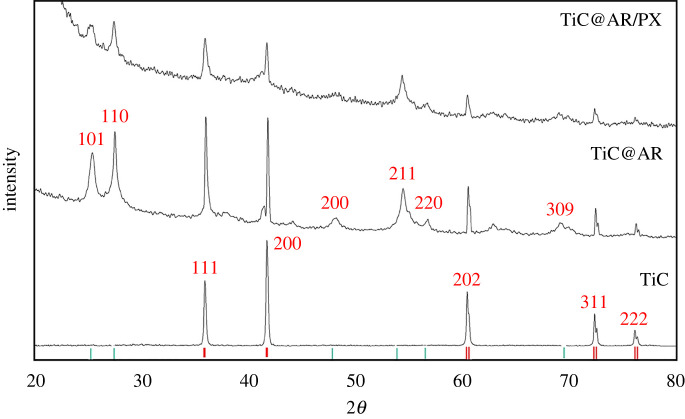
XRD patterns of the samples in different steps.

In addition, XRD analysis was carried out on TiC@AR/PX. For a better comparison of the patterns, the XRD patterns of TiC, TiC@AR and TiC@AR/PX are plotted together (figure 4). After treating with polyvinyl alcohol and xylan, the overall crystallite structure of TiC@AR did not change; however, the peaks became wider and the crystallite size of TiC@AR/PX was reduced to the lowest among the samples (20.5 nm). It shows that the overall structures of TiC and TiO_2_ have not been changed after coating xylan and polyvinyl alcohol on the surface of TiC@AR. Moreover, higher surface activity is expected for TiC@AR/PX due to its smaller crystallite size compared to TiC@AR (34.9 nm).

**Figure 5.**
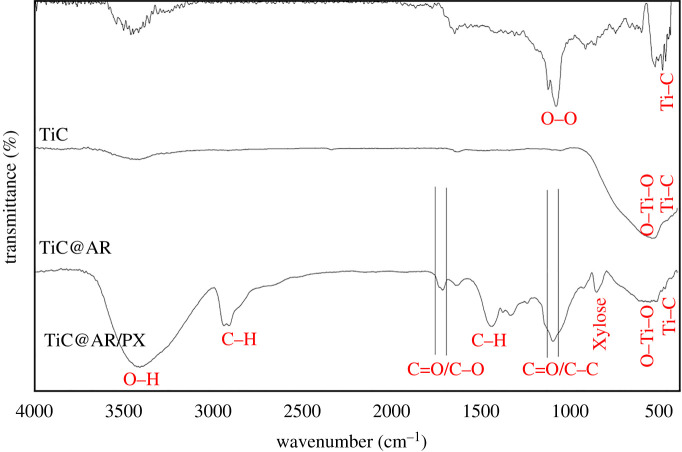
FT-IR spectra of TiC, TiC@AR and TiC@AR/PX.

The surface state of TiC, TiC@AR and TiC@AR/PX was investigated by the FT-IR method in the range 400–4000 cm^−1^ (figure 5).

For a better comparison, the IR spectrum of the as-received TiC powder is included in figure 5. The peak related to the Ti–C vibration can be seen at 465 cm^−1^. The peak corresponding to the oxygen bond is also centred at 1048 cm^−1^. After heat treatment and obtaining TiC@AR, the TiO_2_ peak appeared at around 550–560 cm^−1^. Due to the peak of TiO_2_ and its characterization which is usually wide, the peak related to TiC illustrated itself as a shoulder beside the TiO_2_ peak.

Surface modification of TiC@AR by xylan and polyvinyl alcohol led to the emergence of various organic peaks on the surface of TiC@AR which was converted to a new compound (TiC@AR/PX). Most of the peaks are the same for polyvinyl alcohol and xylan which also overlapped, so there are three regions, including polyvinyl alcohol (**I**), xylan (**II**) and overlapping peaks (**III**). The widest peak is related to the stretching O–H vibration at around 3400 cm^−1^ (**III**). The band at 3000–2800 cm^−1^ corresponds to the stretching vibration of the C–H group (**III**). In addition, there is a sharp double peak between 1750 and 1690 cm^−1^ which can be attributed to C=O and C–O groups (**I**) [34]. Furthermore, the peaks at approximately 1080 cm^−1^ can be assigned to C–O, C–C stretching vibrations (**II**). These peaks belong to the xylose content of xylan. Moreover, there is a tiny sharp peak at approximately 850 cm^−1^, which belongs to *β*-d-pyran-type xylose and ring vibrations [35]. Finally, the peaks related to TiO_2_ and TiC almost remained unchanged compared to TiC@AR.

The result shows that each step has its unique effect by creating proper functional groups. As mentioned in the discussion of the XRD results, the overall crystalline structure of TiC@AR/PX did not change; however, IR analysis confirmed the presence of functional groups of polyvinyl alcohol and xylan on the surface of TiC@AR. Thus, the organic polyvinyl alcohol and xylene were formed on the inorganic anatase and rutile substance. TiC@AR/PX has some advantages such as higher porosity, which is crucial in photon absorption from a light source and in exciting electrons from the valence to the conduction band. On the other hand, the interaction between electrons of the conduction band with the oxygen of the solvent can lead to the formation of radicals with a prominent role in photocatalytic reaction (see §4). Additionally, the peak in the visible range could be due to the xylose (850 cm^−10^) of xylan, implying that TiC@AR/PX could be activated in the visible region.

**Figure 6.**
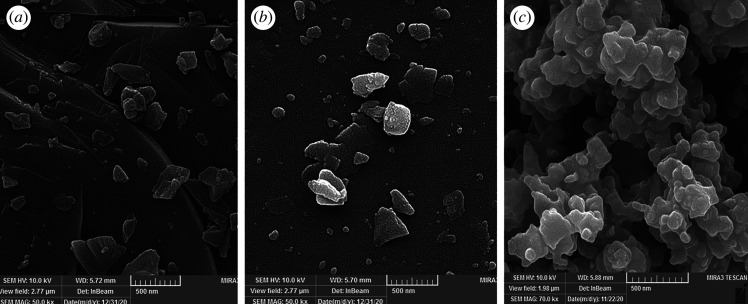
FESEM of TiC (*a*), TiC@AR (*b*), TiC@AR/PX (*c*).

As each crystal facet provides special properties, the morphology of TiC, TiC@AR and TiC@AR/PX was investigated by the FESEM technique (figure 6). After oxidizing TiC and obtaining TiC@AR, the overall morphology of the sample was not changed. However, some white particles appeared on the surface of TiC, which belong to the TiO_2_ phase. The surface modification of TiC@AR by xylan and polyvinyl alcohol made it irregular and cohesive (figure 6*c*). In addition, the appreciable adhesion between the organic (polyvinyl alcohol) and inorganic (TiC@AR/PX) phase enhanced the space between the nanoparticles much larger than their diameter. The average size of TiC is around 55 nm which declined to 40 nm in line with the XRD results. The size of TiC@AR/PX was around 24 nm.

**Figure 7.**
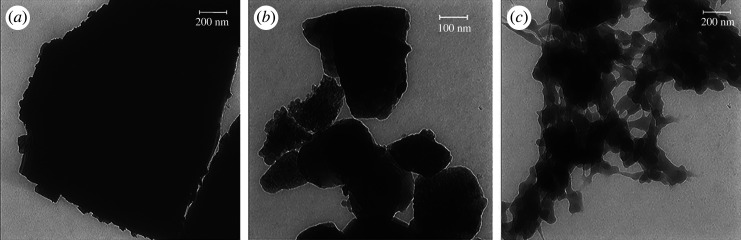
TEM images of (*a*) TiC, (*b*) TiC@AR, (*c*) TiC@AR/PX.

TEM image analysis was carried out for further investigation of the samples as shown in figure 7. TiC has a unique black structure. In the case of TiC@AR, the TiO_2_ layer was formed by increasing the temperature (figure 7*b*). After the formation of the second layer (polyvinyl alcohol and xylan) (figure 7*c*), a polymeric layer was formed around TiC@AR, which promoted the cohesion between different parts as detectable by pale grey colour.

**Figure 8.**
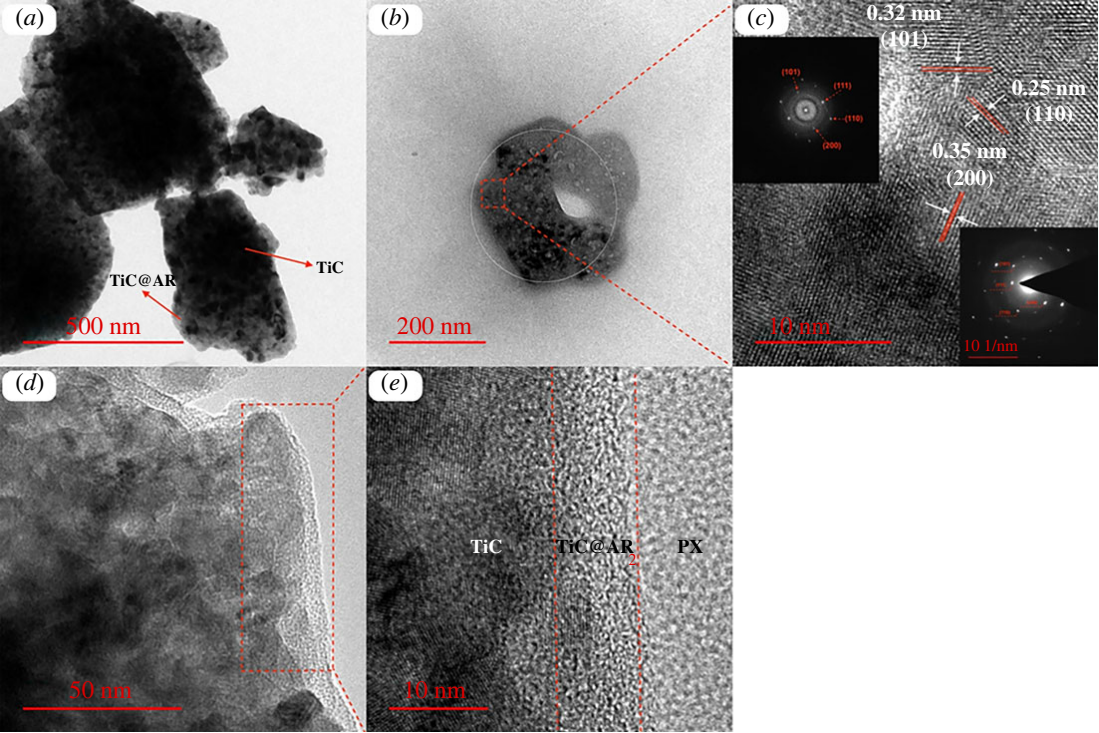
HRTEM images of (*a*–*e*) TiC@AR/PX (selected area electron diffraction image in the inset).

In addition to the TEM analysis, HRTEM has been also carried out (figure 8). According to figure 8*a*, it is obvious that the composite of TiC and TiO_2_ has been obtained. This is also confirmed by figure 8*b*, wherein TiC as black dots are surrounded by TiO_2_ and the mix of the polymers. Based on the selected area electron diffraction pattern (figure 8*c*), the lattice spacing related to TiC (200) and TiO_2_ (110 and 101) is 0.35 nm, 0.25 nm and 0.32 nm, respectively, which is confirmed as being TiC phase (96-901-2565), and anatase–rutile phases (96-900-9087, 96-900-9084). Figure 8*d* is obtained from the edge of the composite, which is clearly in three different patterns (figure 8*e*) and the two last layers belong to the polymeric compounds (polyvinyl alcohol and xylan).

**Figure 9.**
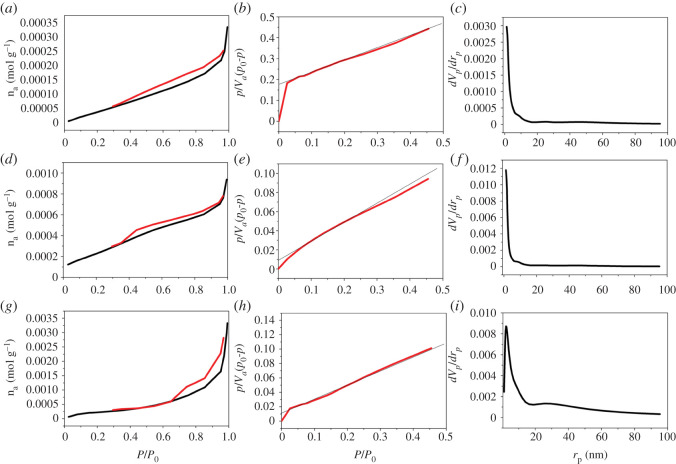
Chemical–physical properties of TiC: (*a*) adsorption/desorption isotherm, (*b*) BET plot, (*c*) BJH plot. TiC@AR: (*d*) adsorption/desorption isotherm, (*e*) BET plot, (*f*) BJH plot. TiC@AR/PX: (*g*) adsorption/desorption isotherm, (*h*) BET plot, (*i*) BJH plot.

The typical N_2_ adsorption–desorption isotherms (BET method) and the pore distribution curves of TiC, TiC@AR and TiC@AR/PX are depicted in figure 9. The micropore volume was investigated in the *P*/*P*_0_ range of 0–0.990 to characterize the type of porosity. The surface activity of the obtained samples was investigated by BET analysis. The BJH method was also applied to determine pore diameter, volume and distribution.

In the case of TiC, the dependency between adsorption–desorption isotherm and pore shape seems to be H3 (IUPAC classification: type III) (figure 9*a*). Nitrogen at 77 K in the relative pressure 0.4–0.45 caused a tensile strength effect which led to a slope related to a force on the hysteresis loop. It has been already confirmed that there is a relationship between the shape of the hysteresis loop and the distribution pattern of a mesoporous compound. The pores of compounds with H3 hysteresis are conical. The pore size is more than 50.0 nm, but according to the result (table 2), the size is much less than 50 nm which is related to the mesoporous size [36], with the classification of V. This implies a weak interaction between the surface of the sample and the adsorbent [37]. Based on this study, the nanoparticles can be also classified as super-nanopore in the size range of 10–100 nm. The overall properties are the same for TiC@AR (figure 9*d*). However, the pores of compounds with H4 hysteresis are slit-shaped. In the case of TiC@AR/PX, the classification is more matched with type IV, suggesting a strong interaction between the surface of TiC@AR/PX and adsorbent (figure 9*g*). The primary part of the isotherm, in the range *P*/*P*_0_ < 0.2, is related to monolayer and multilayer formation and continued the same path corresponding to nonporous solid. The next part of the isotherm, *P*/*P*_0_ > 0.4, illustrated an increased deviation indicating the filling of mesopores (table 2) [38]. The pores in TiC@AR/PX with H4 hysteresis are slit-shaped.

**Table 2.** Physical–chemical properties of the samples.

parameters	TiC	TiC@AR	TiC@AR/PX
BET analysis			
*V*_m_ (cm^3^(STP) g^−1^)	1.3043	4.6399	4.8564
*a*_s,BET_ (m^2^ g^−1^)	5.677	20.195	21.137
*C*	4.4159	28.952	20.022
total pore volume (cm^3^ g^−1^)	0.011	0.031	0.111
mean pore diameter (nm)	8.0006	6.2803	21.111
BJH analysis			
*V*_p_ (cm^3^ g^−1^)	0.012784	0.032953	0.1152
*r*_p,peak_ (area) (nm)	1.21	1.21	2.38
*a*_p_ (m^2^ g^−1^)	7.4141	24.893	30.501
Langmuir plot			
*V*_m_ (cm^3^(STP) g^−1^)	2.7518	5.3164	15.74
*a*_s,Lan_ (m^2^ g^−1^)	11.977	23.139	68.509
*B*	0.02441	0.2975	0.041722

BET analysis was used to determine the surface activity of the samples (figure 9*b,e*,*h*). Based on the result, the surface activity of TiC is 5.67 m^2^ g^−1^ which exhibited a fourfold enhancement (20.195 m^2^ g^−1^) after converting to TiC@AR (table 2). Upon conversion to TiC@AR/PX, the overall surface activity showed no significant changes (21.37 m^2^ g^−1^). Based on figure 8, the maximum adsorption of TiC was 0.00035 n_a_/mol g^−1^, while this parameter was 0.001 and 0.0035 n_a_/mol g^−1^ for TiC@AR and TiC@AR/PX, respectively, implying a step-wise increase in the surface of the samples.

However, significant changes can be seen in the result corresponding to the pores (figure 9*c*,*f*,*i*). While total pores of TiC and TiC@AR are, respectively, 0.011 and 0.031 with three times growth, this parameter became 0.11 cm^3^ g^−1^ for TiC@AR/PX, showing a 10-fold enhancement compared to TiC. This difference is also significant when analysing the mean pore diameter (table 2). The result showed the mesoporosity of all the samples (between 2 and 50 nm) [39]. Based on the hysteresis loop of the samples, both TiC and TiC@AR samples are micro–mesoporous, while TiC@AR/PX showed macroporosity. The data also well matched with the loops (table 2).

The data extracted from BJH determine the significant influence of the conversion (TiC to TiC@AR/PX) process (table 2). The surface activity exhibited an enhancement from 7.41 m^2^ g^−1^ (TiC) to 30.5 m^2^ g^−1^ (TiC@AR/PX).

Two parameters, including adsorbate and adsorbent, affect the adsorption. Based on the Langmuir method, single layer adsorption keeps a few numbers of adsorption sites with the same method and no transmigration of adsorbate in the plane surface. After filling the site, adsorption stopped, implying surface saturation (table 2). The results also confirmed a significant rise in surface activity.

According to the results, the modification by different phases of TiO_2_, polyvinyl alcohol and xylene positively affected the surface activity and porosity, which not only improved light absorption but also provided a better platform for the reaction between electrons of the conduction band and the oxygen of the solvent to form free radicals. In addition, the high porosity of TiC@AR/PX means that electrons react longer whereas one of the main drawbacks of a conventional nano-photocatalyst is its fast recombination rates (a fraction of a second) and termination of the reaction.

**Figure 10.**
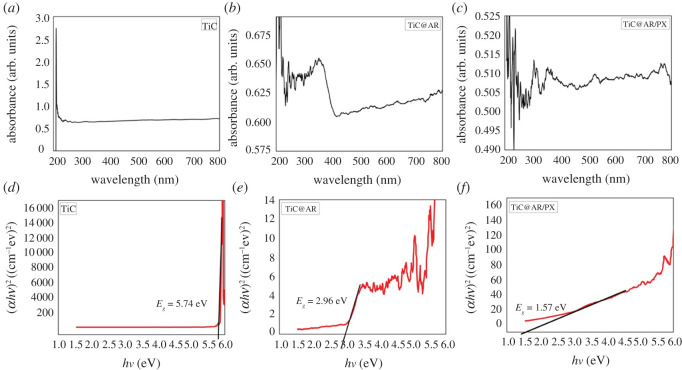
UV–visible spectra of the samples (*a*–*c*); energy bandgap of the samples (*d*–*f*).

The optical activity of photocatalysts is one of the most important factors in their activity within a specific region. DRS spectroscopy was carried out to obtain the optical properties of the samples. However, to have a more accurate and better energy bandgap, DRS spectroscopy was switched to UV–visible spectroscopy by the following equation: −1/[log10(1/R)], where *R* is the reflectance. UV–visible spectroscopy was carried out at 200–800 nm to calculate the energy bandgap of the samples (figure 10).

According to the results, TiC had some sharp peaks between 240 and 290 nm, with the highest peak at 290 nm, implying that the sample cannot absorb visible light (figure 10*a*). On the other hand, TiC@AR showed a peak at 416 nm, suggesting activity in the first region of visible light (violet range) (figure 10*b*). The peaks corresponding to TiC determine the effect of TiC on TiC@AR.

On the other hand, TiC@AR/PX exhibited many peaks in both UV and visible regions, due to the presence of different functional groups of polyvinyl alcohol and xylan (figure 10*c*). To find the best activating region, the Tauc plot was used to find the energy bandgap:

3.1 $$\alpha =\frac{\alpha_{0}(h\nu -E_{g})n}{h\nu },$$

where *α* is the absorption coefficient, *hv* is the photon energy, *α*_0_ and *h* are constants, *E_g_* represents the optical bandgap of the material and *n* is in the range 0.5–3 (depending on the electronic transition).

The energy bandgap was determined from the curves of ${\left(\alpha h\nu \right)}^{2}$ versus *hv*. The *hv* value at the intersection point of the tangent and the *x*-axis shows the bandgap. According to figure 10*d*, the presence of several sharp peaks makes it difficult to determine an accurate place of the dominant peak. Based on the results of UV–visible spectroscopy, the highest peak is located at 220 nm; therefore, it can be claimed that the energy bandgap is around 5.74 eV.

In contrast to TiC, the energy bandgap of TiC@AR can be calculated with more accuracy (figure 10*e*). The obtained graph shows the energy bandgap of 2.96 eV for TiC@AR.

The energy bandgap of TiC@AR/PX was also calculated as 1.57 eV (figure 10*f*), showing a drastic decline. It is worth noting that TiC@AR/PX even showed peaks at longer wavelengths with higher absorption due to possessing different functional groups. Therefore, it can absorb in the IR region of sunlight (accounting for 52% of the sun spectrum intensity). Both TiC and TiC@AR exhibited a decrease in absorption after their highest peak in UV and visible regions. This was the opposite in the case of TiC@AR/PX, and it showed further growth at the longer wavelengths. Another point is the absorption of the samples. Both TiC and TiC@AR have the same absorption between 0 and 45, while this parameter ranged between 80 and 110 for TiC@AR/PX, exhibiting almost a twofold increment.

**Figure 11.**
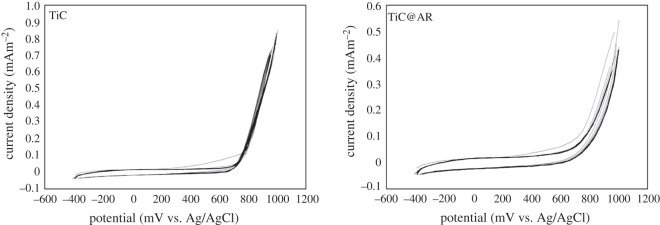
Cyclic voltammetry analysis of TiC and TiC@AR.

Cyclic voltammetry has been done for TiC and TiC@AR to find the position of the valence and conduction bands (figure 11). The potential of the samples can be transformed to the level versus vacuum by the formula *E*_HOMO_ = −(*E*_red_ + 4.8); the factor of 4.8 in the formula is a correction, which is related to Ag/AgCl couple and NHE. Both TiC and TiC@AR showed almost the same behaviour and the oxidation peak appeared around 0.7 V so the *E*_HOMO_ is obtained as −5.5 V versus vacuum and consequently the potential of the samples is 1.1 V versus NHE through the formula of *φ*_LUMO_ = −[*E*_HOMO_ − (−4.4)].

## 4. Photocatalytic activity of the samples

**Figure 12.**
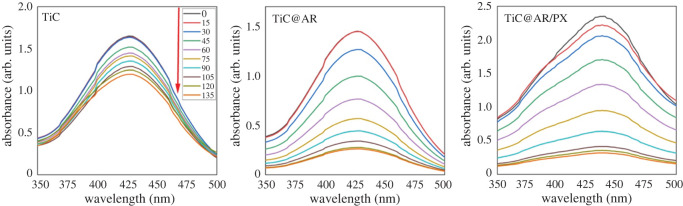
Comparison of the reduction in concentration by visible light.

The photocatalytic activity of the samples was tested using BPB dye (figure 12). The electrons (e^−^) located in the valence band can be excited by a light source and transferred to the conduction band, leaving holes (h^+^) in the valence band. Electron–hole pairs promote photocatalytic degradation.

**Figure 13.**
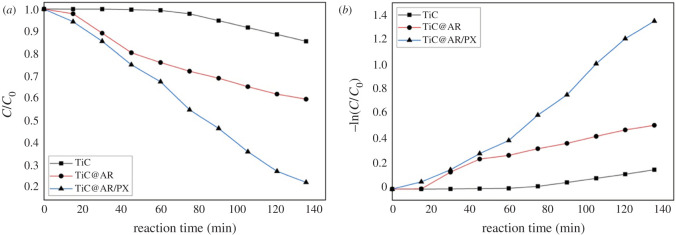
Bromophenol blue removal under visible light: (*a*) comparing the efficiency of decomposition by different samples under visible light and (*b*) photocatalytic degradation kinetics of the samples under visible light.

According to the results, TiC showed better degradation (15%) (table 3). After heat treatment and the formation of the TiO_2_ phase on TiC (TiC@AR), the photocatalytic activity of TiC@AR increased insignificantly (40%). Upon modifying with polyvinyl alcohol and xylan (TiC@AR/PX), a remarkable enhancement occurred in photodegradation of BPB (2 times more than TiC@AR) (75%) (figure 13*a*).

**Table 3.** Photocatalytic features of the samples.

sample	degradation (%)	first-order rate		real rate	
		*K*_obs_ (min^−1^)	*R*^2^	*K*_obs_ (min^−1^)	*R*^2^
TiC	15	0.0011	0.83328	1 × 10^−5^x^2^	0.9927
TiC@AR	40	0.004	0.96817	1 × 10^−5^x^2^	0.9819
TiC@AR/PX	75	0.0106	0.97507	4 × 10^−5^x^2^	0.9958

The *K*_obs_ values are presented in table 3 and figure 13*b*. The real degradation kinetics cannot be regarded as a first-order rate (ln(*C*_0_/*C*) = *K*_obs_*t*; in which *K*_obs_ is the rate constant (min^−1^), *t* is the reaction time (min), *C*_0_ is the aqueous concentration of BPB before the degradation and *C* is the concentration of BPB at different times after degradation). The best explanation for the degradation rate of each sample can be found in table 3, including the second-order kinetics for TiC, TiC@AR and TiC@AR/PX with an accuracy of more than 98%. However, to simplify the calculations, the rate of degradation was measured as the pseudo-first-order rate. This rate is almost acceptable for TiC@AR and TiC@AR/PX with an accuracy above 97% (table 3). In any mode, the value of *K* increased from TiC to TiC@AR/PX (around five times).

Energy bandgap and surface activity play important roles in the degradation of BPB. After heat treatment of TiC and obtaining TiC@AR the energy bandgap reached 2.96 eV which is in the violet region. After modification by polyvinyl alcohol and xylan, it remarkably reduced to 1.57 eV (IR region). On the other hand, the surface activity showed a fourfold enhancement after TiC conversion into TiC@AR; upon modifying by polymers, it did not change dramatically. The significant difference in the BPB degradation ability of TiC@AR/PX and two other compounds can be attributed to its energy bandgap. However, the result of the Langmuir model illustrates that the surface activity of the first layer is also involved. Based on the Langmuir result, the surface activity of TiC@AR/PX (68.5 m^2^ g^−1^) is above six times more than TiC (11.9 m^2^ g^−1^) and two times more than TiC@AR (23.13 m^2^ g^−1^).

## 5. The mechanism of the reaction

**Figure 14.**
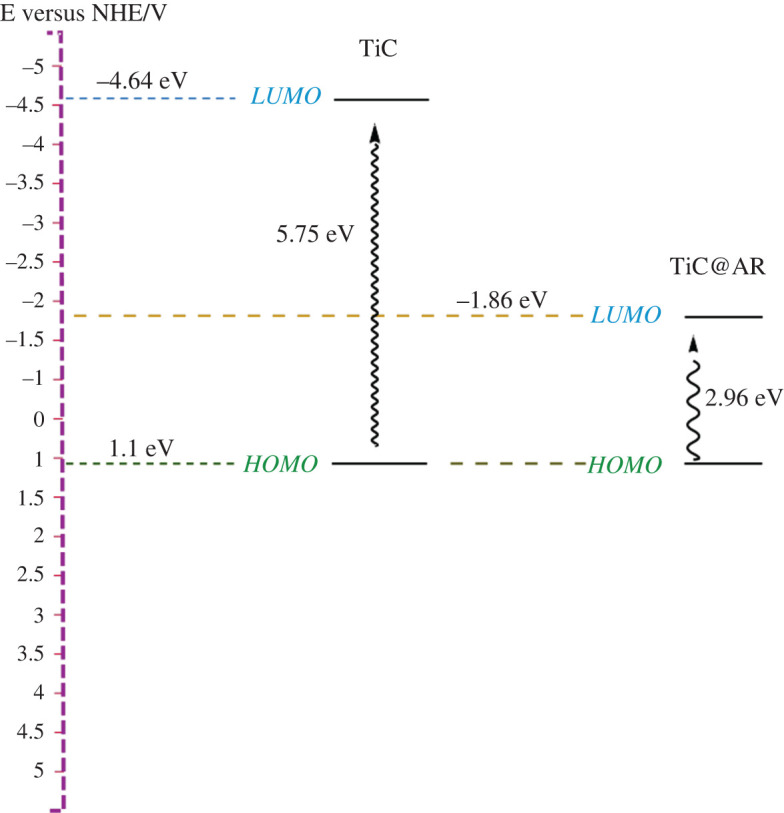
Schematic diagram of redox potentials of TiC and TiC@AR.

**Figure 15.**
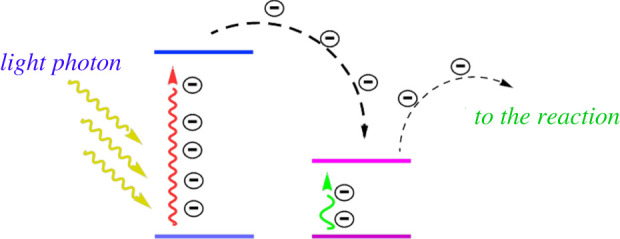
Proposed electron transfer route.

**Figure 16.**
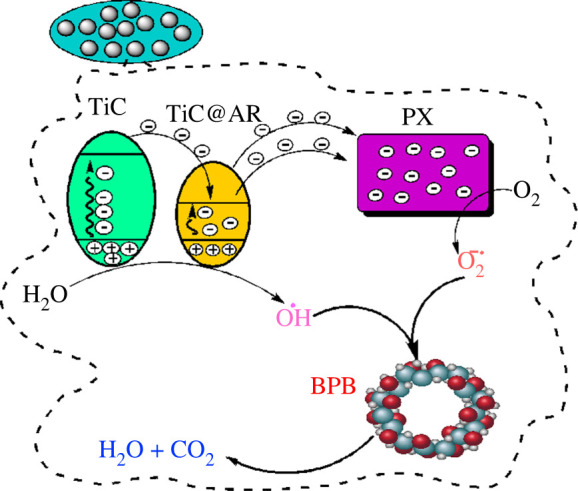
Proposed mechanism for the degradation of bromophenol blue.

The following mechanism can be assumed for the degradation process of BPB. According to the DRS analysis, the energy bandgaps of TiC and TiC@AR are 5.74 eV and 2.96 eV, respectively, and the valence band of both samples is at 1.1 V so the conduction bands of TiC and TiC@AR are located at −4.64 and −1.86 eV, respectively (figure 14). Based on the mentioned reasons, the movement and the direction of electrons can be proposed as shown in figure 15. The next step is migration of electrons to the polymers (polyvinyl alcohol and xylan). Polymers have a very important role to accumulate and then accelerate the trapped electrons to the O_2_ existing in the surrounded environment and form O_2_^−^ radicals [40]. On the other hand, holes generated in the valence bands react with H_2_O and form hydroxyl radicals (HO^.^). Finally, both radical agents react with BPB to reduce its concentration and produce H_2_O and CO_2_ (figure 16).

## 6. Conclusion

In this study, TiC@AR was synthesized through the heat treatment of TiC. TiC@AR/PX was obtained by polymerization of TiC@AR using xylan and polyvinyl alcohol. The heat treatment was set at different temperatures (350°C, 400°C and 450°C) and times (30, 60, 90, 120, 180 and 240 min) to find the optimal condition to achieve the best TiO_2_ with both anatase and rutile, on the TiC phase.

The energy bandgap was reduced to visible (TiC@AR) and IR (TiC@AR/PX) ranges, while the surface activity exhibited a sixfold increase. These parameters made TiC@AR/PX a powerful platform to reduce the concentration of BPB dye (by approx. 75%) under sunlight, which is a promising method in the photocatalytic field.

## Data Availability

In the paper, we present new data and the instructions how to reproduce the experiments are fully explained in the main text of the paper and electronic supplementary material [41]. Datasets for all figures are available from the Dryad Digital Repository: https://datadryad.org/stash/share/BMOegp37s2NfUma_7QMIyITUFGJp3hck0Eb9iTuT20s [42].
